# Current Advances in Gene Therapies of Genetic Auditory Neuropathy Spectrum Disorder

**DOI:** 10.3390/jcm12030738

**Published:** 2023-01-17

**Authors:** Anissa Rym Saidia, Jérôme Ruel, Amel Bahloul, Benjamin Chaix, Frédéric Venail, Jing Wang

**Affiliations:** 1 Institute for Neurosciences of Montpellier (INM), University Montpellier, INSERM, 34295 Montpellier, France; 2 Cognitive Neuroscience Laboratory, Aix-Marseille University, CNRS, UMR 7291, 13331 Marseille, France; 3 Department of ENT and Head and Neck Surgery, University Hospital of Montpellier, 34295 Montpellier, France

**Keywords:** gene therapy, auditory neuropathy, auditory synaptopathy, hidden hearing loss, genetic deafness, hearing restoration

## Abstract

Auditory neuropathy spectrum disorder (ANSD) refers to a range of hearing impairments characterized by an impaired transmission of sound from the cochlea to the brain. This defect can be due to a lesion or defect in the inner hair cell (IHC), IHC ribbon synapse (e.g., pre-synaptic release of glutamate), postsynaptic terminals of the spiral ganglion neurons, or demyelination and axonal loss within the auditory nerve. To date, the only clinical treatment options for ANSD are hearing aids and cochlear implantation. However, despite the advances in hearing-aid and cochlear-implant technologies, the quality of perceived sound still cannot match that of the normal ear. Recent advanced genetic diagnostics and clinical audiology made it possible to identify the precise site of a lesion and to characterize the specific disease mechanisms of ANSD, thus bringing renewed hope to the treatment or prevention of auditory neurodegeneration. Moreover, genetic routes involving the replacement or corrective editing of mutant sequences or defected genes to repair damaged cells for the future restoration of hearing in deaf people are showing promise. In this review, we provide an update on recent discoveries in the molecular pathophysiology of genetic lesions, auditory synaptopathy and neuropathy, and gene-therapy research towards hearing restoration in rodent models and in clinical trials.

## 1. Introduction

**Figure 1:**
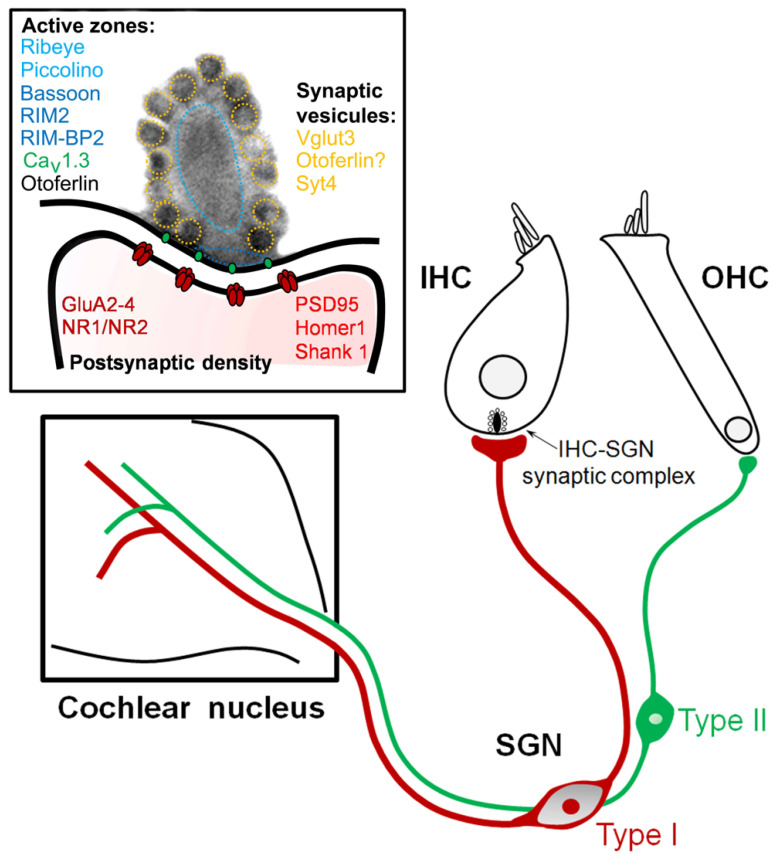
Inner hair cell (IHC)–spiral ganglion synaptic complex. The IHC is connected to all type I spiral ganglion neurons (SGNs) forming the radial afferent system (red) going to the cochlear nuclei. The OHC synapses with small endings from type II spiral ganglion neurons, forming the spiral afferent system (green). Molecular composition of a mature ribbon of the inner hair–cell synaptic complex ensuring the temporal precision of peripheral sound encoding. The mature ribbon synapse between the sensory inner hair cells (IHCs) and postsynaptic spiral ganglion neurons (SGNs) involves the spatial confinement of several molecular components: presynaptic density merge to one single ribbon anchor and, postsynaptically, one continuous elongated postsynaptic density composed by functional synaptic AMPA-preferring glutamate receptors, but also some silent NMDA receptors. Font color indicates association with the correspondingly colored pre-/postsynaptic localization.

Hearing in mammals relies on the ability of the sensory hair cells to convert sound-evoked mechanical stimuli into electrochemical signals. The hair-bundle deflection induces rapid opening of sensory transduction channels, leading to the generation of an influx of cations into the IHC. This results in a depolarization potential, allowing an influx of calcium through voltage-dependent calcium channels. The coupling of Ca^2+^ channels at the presynaptic site of the ribbon synapse triggers high-rate synaptic vesicle fusion and the release of neurotransmitter glutamate from the synaptic cleft. The release of glutamate in the synapse activates Ca^2+^-sensitive AMPA receptors (Figure 1). This initiates the generation of neural spikes in spiral ganglion neuron (SGN) fibers, which encodes information about sound stimuli that is sent to the central nervous system. A dysfunction at any level of this complex transduction machinery may disturb the coding of acoustic features, particularly of temporal cues. The potential sites of damage are diverse, including the IHCs, IHC ribbon synapses, or synaptopathy, (e.g., pre-synaptic release of glutamate or postsynaptic terminals dendrites of the spiral ganglion neurons), or can be due to demyelination and axonal loss of the auditory nerve fibers and their targets in the cochlear nucleus (i.e., neuropathy, Figure 1). These auditory pathologies are named auditory neuropathy spectrum disorder (ANSD), in which the activity of outer hair cells (OHCs) is maintained (Figure 1) [1,2,3,4].

**Figure 2:**
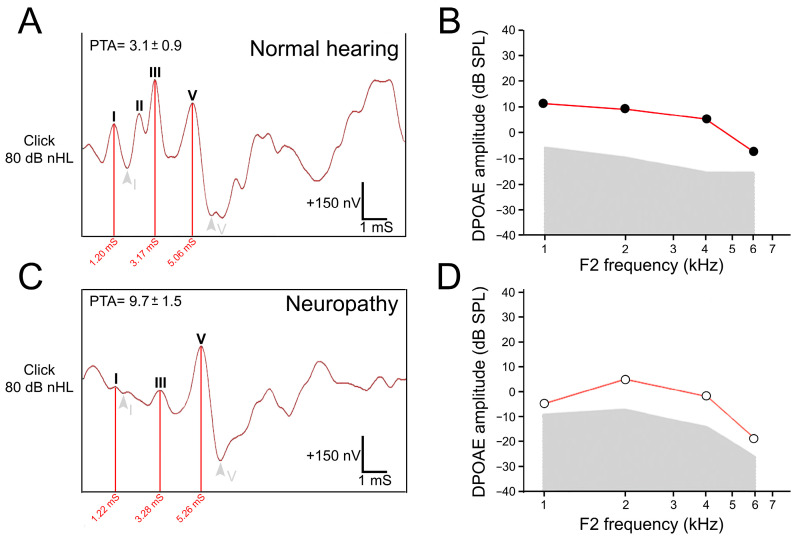
Auditory brainstem responses (ABRs) and distortion product of otoacoustic emissions (DPOAEs) recorded from one normal hearing control (**A**,**B**) and one patient with speech understanding difficulties (**C**,**D**). (**A**) ABRs were recorded at 80 dB nHL (decibels normal hearing level) with normal amplitudes, latencies, and morphology. (**B**) No abnormalities were detected in the DPOAEs. (**C**) A patient with speech understanding difficulties showed a clear decrease in the amplitude of waves I, II, and III. The I-V interval was 4.04 vs. 3.86 for the patient and a normal hearing subject, respectively. (**D**) DPOAEs were still present in this patient. PTAs: mean of pure-tone audiometry thresholds recorded at 250, 500, 1000, 2000, 3000, and 4000 Hz. Note that PTA is within the normal range in the patient with speech understanding difficulties. The audiologic results of this patient suggest a hidden auditory neuropathy.

The clinical profiles of ANSD are quite heterogeneous, depending on the variety of etiologies. ANSD can result from syndromic and non-syndromic genetic abnormalities, as well as environmental causes (e.g., hypoxia, noise-exposure, cytotoxic oncologic drugs) and aging. ANSD is one of the common causes of hearing loss, affecting between 1.2% and 10% of those with hearing loss [5]. Audiologically, ANSD is characterized by mild to profound sensory neural hearing loss, with impaired or absent compound action potentials (CAP) and auditory brainstem responses (ABRs, Figure 2) and deteriorated speech audiometry in quiet [6]; these are associated with normal otoacoustic emissions (OAE, Figure 2) or cochlear microphonics (CM), indicating normal OHC function. Additionally, the absence of the middle-ear stapedial reflex and of the contralateral suppression of otoacoustic emissions are usually observed [1,5,7,8].

Electrocochleography (ECochG) and tests of neural adaptation remain a powerful diagnostic tool to help identify the site of a lesion. For example, the absence of the summating potential in ECochG indicates the loss of IHC mechanoelectrical transduction or of the IHCs themselves. Furthermore, ECochG can also be used to distinguish auditory synaptopathy from auditory neuropathy. Indeed, patients with auditory synaptopathy displayed enhanced adaptation to frequency specific sounds [9]. By contrast, patients with auditory neuropathy showed normal adaptation for low-frequency sounds but abnormally enhanced adaptation to high-frequency sounds [9]. In patients with auditory synaptopathy, abnormal loudness adaptation is likely related to the disorder affecting IHC-ribbon synapses in the organ of Corti along the basilar membrane [9]. In the cases of auditory neuropathy, loss of nerve fibers is equally distributed throughout the cochlea, and thus the observed neural conduction disorder is probably independent of the origin of the fibers along the basilar membrane [2]. The normal adaptation observed in the low-frequency region is therefore unexpected and may reflect the compensation by central auditory structures involved in loudness perception, reducing the auditory nerve input in a frequency-specific manner [10,11].

Hidden hearing loss (HHL) or supraliminal hearing disorders are probably a specific type of ANSD caused by, e.g., noise exposure, aging, or peripheral neuropathy and characterized by normal pure-tone hearing thresholds together with deficits in sound-evoked auditory nerve activity (Figure 2). Patients with HHL display normal speech audiometry thresholds in quiet, well-synchronized ABRs, but with impaired speech discrimination in noisy environments [12].

Currently, clinical options for the hearing rehabilitation of patients suffering from ANSD are hearing aids that can amplify sound for mild or moderate deafness, or cochlear implants for severe deafness [12]. The advantage of the latter is that they can bypass non-functional sensory hair cells by directly stimulating the remnant auditory neural structures within the deafened cochlea. Actually, the data published in the literature on the long-time outcomes of hearing rehabilitation with hearing aids in children with ANSD are partially contradictory [13,14,15]. Some report good hearing rehabilitation [13,14,15], while others describe a lack of hearing and communication benefits from hearing aids in children with ANSD [1,16]. Cochlear implants, which electronically stimulate the SGNs, might provide effective auditory rehabilitation for patients with auditory synaptopathy because the generation and propagation of spikes is maintained. Generally, patients with lesions affecting the auditory nerve show poor performance with cochlear implants, probably due to altered neural transmission of the electrical signal from the cochlear implant [12]. However, in patients with OPA1-related auditory neuropathy one year after implantation, improvement in speech perception and synchronous activation of auditory pathways was observed [17], probably by either bypassing the site of lesion (which could be located in the terminal dendrites) and/or by the electrical stimuli inducing well-defined temporal SGN activation. However, to date, data on the outcome of auditory neuropathy rehabilitation are limited [5,18].

The past decade has seen significant advances in the understanding of the molecular pathogenic mechanisms that contribute to hearing impairments induced by environmental and genetic factors. This, in turn, has brought renewed hope to concepts of replacing or correcting the mutant sequences or defected genes in order to prevent auditory neurodegeneration or to promote regeneration of auditory synapse and nerve fibers. This review begins by outlining our current understanding of the molecular pathways that mediate genetic ANSD. The following sections discuss recent discoveries in gene therapies using newly designed genetic therapeutic tools for replacing the mutant sequences or defected genes for restoring hearing by rescuing cochlear function through the regeneration of synapses and/or auditory nerve fibers. These tools provide promising perspectives for the future restoration of hearing in deaf people.

## 2. Pathogenic Mechanisms of Auditory Neuropathy

Syndromic auditory neuropathy affects multiple cranial and peripheral nerves, while non-syndromic auditory neuropathies are limited to the auditory nerve. Most cases of non-syndromic auditory neuropathy result from impaired synaptic transfer [5].

### 2.1. Non-Syndromic Auditory Synaptopathies

Genetic auditory synaptopathies generally only cause deafness, such as the mutations in the *CACNA1D* gene encoding the Cav1.3L-type Ca^2+^ channel, the *OTOF* gene encoding Otoferlin, the *SLC17A8* gene encoding Vglut3, or the *DIAPH3* gene encoding the diaphanous formin 3.

#### 2.1.1. Otoferlin-DFNB9

The OTOF gene encodes otoferlin, which is a critical calcium sensor for synaptic exocytosis in cochlear IHCs [19,20]. Otoferlin is also involved in vesicular reformation, re-supply, and tethering at the active zone, making otoferlin a multi-tasking protein [20,21]. Mutations in the gene encoding otoferlin are responsible for autosomal recessive profound prelingual deafness, DFNB9 [22]. To date, about 220 pathogenic variants in *OTOF* have been identified [23]. The majority of these mutations are assumed to be nonsense or truncation mutations that provoke the inactivation of otoferlin [24]. Patients with variants in *OTOF* displayed milder hearing loss, as well as progressive and temperature-sensitive hearing loss, while OAEs were preserved [22,25,26,27]. Children harboring biallelic mutations of the *OTOF* gene displayed profound hearing loss, absence of ABRs and CAP, but preservation of DPOAEs and the amplitude of CM [28]. Otoferlin knock-out mice, which are profoundly deaf due to a failure of sound-evoked neurotransmitter release at the IHC synapse, are likely to be an appropriate animal model for DFNB9 [29,30]. In these mice, Ca^2+^-triggered exocytosis in IHCs is almost abolished [29,30]; synaptic vesicles were found near the membrane at the active zone, suggesting that an absence of vesicles did not limit signal transduction, but that a late step of exocytosis was disrupted [30].

#### 2.1.2. VGLUT3-DFNA25

Vesicular glutamate transporters (VGLUTs) are responsible for glutamate loading into synaptic vesicles, which is essential in order to achieve synaptic transmission [31]. VGLUT3 is expressed in small subsets of neurons in the central nervous system [31,32]. In mice, VGLUT3 is expressed in the IHCs [33,34] and the OHCs [35]. The genetic ablation of *Slc17a8* in mice results in the absence of CAP or ABRs to acoustic stimuli, while ABRs could be elicited by electrical stimuli, and robust otoacoustic emissions were recorded in these mice [33,34]. This thus reflects a failure in activation of the ascending auditory pathway, while the activity in OHCs is unaffected [33,34,36,37]. Patients with a 12q22-q24 deletion in the *SLC17A8* gene at the DFNA25 locus display congenital and non-syndromic autosomal dominant deafness [33,38,39]. The deafness in patients was characterized as high-frequency, progressive sensorineural hearing loss, with good hearing rescue through cochlear implantation, thus reinforcing the hypothesis of synaptopathy [33,39]. *VGLUT3*^A224V/A224V^ mice harboring the p.A221V mutation (p.A221V in humans corresponds to p.A224V in mice) in the *Slc17a8* gene displayed progressive hearing loss with intact OHC function [40]. The summating potential was, however, reduced, indicating the alteration of the IHC receptor potential. Scanning electron microscopy examinations revealed the collapse of IHC stereocilia bundles, leaving those from OHCs unaffected. In addition, IHC ribbon synapses underwent structural and functional modifications at later stages. These results suggest that DFNA25 stems from a failure in mechano-transduction followed by a change in synaptic transmission [40].

#### 2.1.3. Cav1.3-SANDD

Calcium influx at the base of the IHCs near the ribbon synapse is mediated via the L-type calcium (Ca^2+^) channel Cav1.3, which is the main voltage-gated Ca^2+^ channel in IHCs and essential for hearing. Cav1.3 translates sound-induced depolarization into neurotransmitter glutamate release at the synaptic site, resulting in signal transmission to the auditory nerve [41]. Cav1.3-encoding by the *CACNA1D* gene is widely distributed across different cells such as OHCs, IHCs, cardiomyocytes, neuroendocrine cells, and neurons. A Cav1.3. mutation in *CACNA1D* may cause both sinoatrial node dysfunction and deafness (termed SANDD syndrome) in mice and in humans, in humans closely resembling that of *Cacna1d^−/−^* mice [41,42]. Ca_v_1.3 is required for normal hearing and cardiac pace making in humans, and loss of function in only a subset of channels is sufficient to cause SANDD syndrome [42]. Loss-of-function mutations in the *CACNA1D* gene causes impaired synaptic neurotransmission at the IHC ribbon synapse in KO mice [41,43]. Cav1.3 protects the sensory hair cells during cochlear aging through reducing calcium-mediated oxidative stress in C57BL/6J male mice [44] and plays important roles in inner ear differentiation [45].

#### 2.1.4. CABP2-DFNB93

Calcium-binding protein 2 (CABP2) is a potent modulator of IHC voltage-gated calcium channels CaV1.3. CABP2 regulates Ca^2+^ influx at the presynaptic site [46,47] and thus also the vesicular release of glutamate. Pathologic mutations in *CABP2* lead to autosomal-recessive, moderate-to-severe non-syndromic hearing impairment DFNB93 [48,49,50,51]. DFNB93 patients displayed an auditory synaptopathy phenotype with normal OAEs [52]. Using a knock-out mouse model, Picher et al. [52] demonstrated that DFNB93 hearing impairment may result from an enhanced steady-state inactivation of CaV1.3 channels at the IHC synapse, thus limiting their availability to trigger synaptic transmission, resulting in elevated auditory thresholds [52]. This, however, does not seem to interfere with cochlear development and does not cause the early degeneration of hair cells or their synaptic complex [52,53]. These results suggested an extended window for gene therapy.

#### 2.1.5. DIAPH3-AUNA1

Auditory neuropathy, non-syndromic, autosomal dominant 1 (AUNA1) is a form of delayed-onset, progressive human deafness resulting from a point mutation in the 5′ untranslated region of the Diaphanous homolog 3 (*DIAPH3*) gene. The *DIAPH3* mutation leads to the overexpression of the DIAPH3 protein, a formin family member involved in cytoskeleton dynamics [54]. Patients with AUNA1 displayed absent or altered ABR, while OHC functions are still maintained [1,55], thus indicating auditory neuropathy. Transgenic mice overexpressing Diap3 exhibit a progressive threshold shift but maintained a distortion product of otoacoustic emissions (DPOAEs) [54,56]. Morphological assessments revealed a selective and early onset alteration of the IHC cuticular plate and fused stereocilia with the eventual loss of the capacity of IHC to transmit incoming sensory stimuli [54,56]. Furthermore, a significant reduction in the number of IHC ribbon synapses was observed over 24 weeks in mutant mice, although this reduction did not correlate temporally with the onset and progression of hearing loss or of stereocilia bundle anomalies [54]. Together, these results suggest an important function of Diap3 in regulating the assembly and/or maintenance of actin filaments in IHC stereocilia, as well as a potential role at the IHC ribbon synapse.

### 2.2. Syndromic Auditory Neuropathy

Genetic neuropathies frequently affect other neurons, thus leading to syndromic phenotypes such as Charcot–Marie–Tooth disease, autosomal dominant optic atrophy, Leber’s hereditary optic neuropathy, Friedreich’s ataxia, Mohr–Tranebjaerg syndrome, Refsum disease, or Wolfram syndrome [57,58,59,60].

#### 2.2.1. Charcot–Marie–Tooth

Autosomal-dominant Charcot–Marie–Tooth (CMT) is the most common hereditary peripheral polyneuropathy characterized by the degeneration of peripheral nerves. CMT can be classified into two major categories: TMC type 1 (demyelinating neuropathies) and type 2 (axonal form of neuropathies) [61,62]. CMT patients carry mutations in the MPZ genes for myelin protein zero or PMP22 coding for proteins essential for the formation and adhesion of myelin [2,63,64]. CMT type 1 A (CMT1A) is the predominant subtype, which is a demyelinating peripheral neuropathy characterized by distal muscle weakness, sensory loss, areflexia, and slow motor- and sensory-nerve conduction velocities [1,62,63]. Hearing impairment is also a relatively common symptom of CMT1A. Compared to controls, CMT1A patients had a significantly decreased speech perception capacity in a noisy environment, as well as decreased temporal and spectral resolution, thus suggesting that demyelination of auditory-nerve fibers in CMT1A causes defective cochlear neurotransmission [65]. Patients with CMT type 1 and 2 showed a delayed or reduced amplitude ABR, as well as an impaired speech intelligibility, which are electrophysiological evidence of auditory neuropathy [62].

#### 2.2.2. Autosomal-Dominant Optic Atrophy

Autosomal-dominant optic atrophy (DOA) is the most frequent form of hereditary optic neuropathy [66], with a reported frequency of 1:10,000, and is caused by heterozygous variants in the *OPA1* gene encoding a mitochondrial-dynamin-related large GTPase [67,68,69]. OPA1 is involved in many mitochondrial functions, notably in the maintenance of the respiratory chain and cell membrane potential [70,71,72], cristae organization, control of apoptosis [72,73], and mitochondrial DNA maintenance [74,75,76]. DOA was initially described as a non-syndromic moderate-to-severe loss of visual acuity, with an insidious onset in early childhood caused by a progressive loss of retinal ganglion cells [77]. In the last decade, the clinical spectrum of DOA has been extended to a wide variety of symptoms, including deafness, ataxia, neuropathy, and myopathy, and is now called dominant optic atrophy plus (DOA*plus*) [74,78,79]. Deafness is the second-most prevalent clinical feature in DOA*plus*, affecting about 20% of all DOA patients [17,74,78,79,80].

The association of DOA and deafness is classically related to the R445H mutation in exon 14, but other *OPA1* mis-sense variants have already been reported in the literature [79,81]. Here, hearing loss starts in childhood or early adulthood [79,82]. Although the majority of studies broadly qualify the hearing disorder as ‘sensorineural hearing loss’, some authors have proposed auditory neuropathy as the pathophysiological mechanism underlying the hearing impairment in OPA1-DOA [17,70,83,84]. Audiolological examination of OPA1 hearing impaired patients harboring missense mutations showed impaired speech perception and absence or profound alteration of ABRs but preservation of OAE and even enhanced CM potentials reflecting normal OHC function [28].

#### 2.2.3. Leber Hereditary Optic Neuropathy

Leber hereditary optic neuropathy (LHON) is the most common mitochondrial genetic disease. It is characterized by bilateral, subacute, painless loss of vision, and over 95% of LHON cases are caused by one of three mitochondrial DNA (mtDNA) point mutations: 3460G>A, 11778G>A, and 14484T>C or mutation in the *TMEM126A* gene coding a mitochondrial protein. Severe axonal degeneration with demyelination of the optic nerve had been indicated by histological necropsy studies [85]. Patients with Leber hereditary optic neuropathy also show signs of auditory neuropathy [86,87].

#### 2.2.4. Friedreich’s Ataxia

Friedreich’s ataxia (FRDA) is the most frequent autosomal-recessive inherited ataxia caused by mutations in the FXN gene coding for the mitochondrial protein Frataxin involved in regulating iron accumulation in the mitochondria. FRDA is due to an abnormal repetition of the GAA triplet (100 to 2000 GAA triplets) in the *FXN* gene [88]. In addition to impaired balance and coordination of voluntary movements, Friedreich’s ataxia is associated with hearing impairment, including difficulty understanding speech in background noise; auditory thresholds were, however, unchanged [88,89,90,91], nor was OHC function [92]. Most affected individuals show abnormalities in auditory neural and brainstem responses as a result of auditory neuropathy [92,93,94]. Of FRDA patients, 8 to 13% show sensorineural hearing loss, as revealed in a pure-tone audiogram [95].

#### 2.2.5. Mohr–Tranebjaerg Syndrome

Mohr–Tranebjaerg syndrome, in which deafness with progressive dystonia and visual impairment are associated, can be classified as a non-isolated auditory neuropathy. Indeed, observation of post-mortem samples shows neuronal loss with preservation of OHCs [96]. Here, again, mutations (DDP1 for deafness-dystonia) of TIMM8A/DDP1, which codes for a polypeptide of 97 amino acids located in the mitochondria, are at the origin of this syndrome.

## 3. Gene Therapies for Genetic Synaptopathies and Neuropathies

Gene therapy is an experimental technique that uses genes to treat or prevent disease by introducing a desired foreign gene or gene-regulatory element, such as RNA interference, into the target cells to replace or repair the defective gene [97]. Future gene therapy could promise the restoration of hearing in some forms of monogenic deafness where cochlear morphology is preserved for a period of time that allows intervention to restore hearing. Several viral vectors (e.g., adenovirus (Ad), adeno-associated virus (AAV), lentivirus) have already been used to transduce the inner ear [98,99]. The most recent studies have focused on optimizing AAV-based vector systems (Table 1), due to their efficiency in transducing cells of the sensory epithelium.

### 3.1. Restoration of Neurotransmission in IHC Synapses

#### 3.1.1. DFNB9

Otoferlin knock-out mice, which are profoundly deaf due to a failure of sound-evoked neurotransmitter release at the IHC synapse, are likely to be an appropriate animal model for DFNB9 [29,30]. AAV-mediated gene transfer of the gene encoding otoferlin is technically challenging, due to the limited DNA packaging capacity of AAVs (≈4.7 kb). This limit makes it impossible to package large genes such as *Otof* (cDNA ~6 kb). To overcome this problem, Reisinger et al. [100] investigated the possibility of restoring hearing using the AAV-mediated gene transfer of the synaptotagmin1 gene into mouse hair cells deficient in otoferlin. Up to the fourth post-natal day in the mouse, calcium-triggered exocytosis depends on synaptotagmin 1 [101], whereas synaptotagmin1/2 are not expressed in adult IHCs [102]. Thus, extending synaptotagmin 1 expression over a longer term may rescue the loss of otoferlin in DFNB9. Unfortunately, the strategy failed to restore the Ca^2+^ influx-triggered exocytosis in IHCs of *Otof^−to^* mice [100].

Akil et al. [103] adapted a reported dual AAV-vector method for the delivery of large cDNAs [104]. They used two different recombinant vectors, one containing the 5′ and the other the 3′ portions. They showed that a single delivery of the two vectors through the round-window membrane into the cochlea of *Otof*^−/−^ mutant mice on P10, P17, or P30 restored production of the full-length protein and partially restored hearing in deaf *Otof*^−/−^ mice [103].

**Figure 3:**
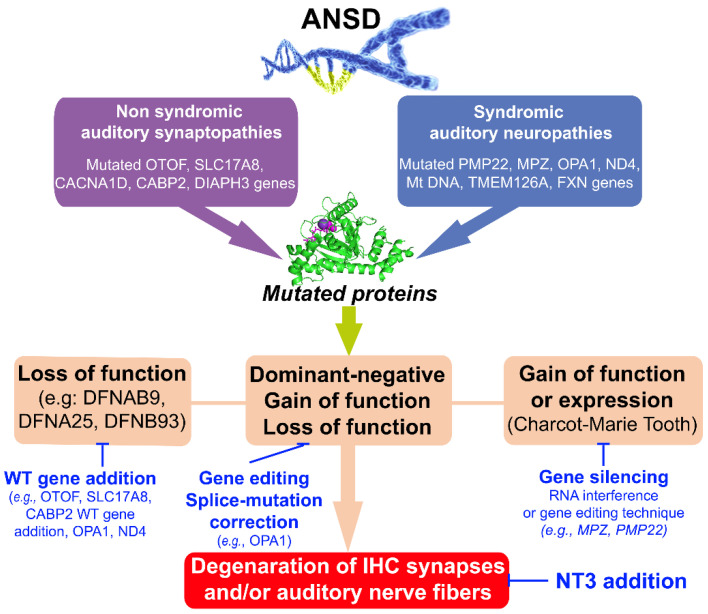
Summary diagram of the design gene therapies discussed in this review. Genetic syndromic auditory neuropathy affects multiple nerves, while non-syndromic auditory synaptopathy is limited to IHC ribbon synapses. These auditory phenotypes may result from loss of function, dominant negative effects, or gain-of-function expression mechanisms. Hearing rescue strategies may be designed to target the different levels of disease mechanisms, i.e., (i) WT gene addition to rescue loss function phenotype; (ii) gene or mRNA editing to correct dominant-negative, gain-of-function, or loss of function mutations; (iii) gene silencing to correct gain-of-function or expression; and (iv) NT3 gene or protein addition to enhance the repair and/or regeneration of auditory synapses and nerve fibers. The mutated protein shown here is the OPA1 protein involved in dominant optic atrophy (DOA).

More recently, Rankovic et al. [105] used a new gene therapeutic method of overloaded AAVs for packaging the full-length *Otof*. Indeed, they packaged the full-length *Otof* into several naturally occurring as well as more recently developed and highly potent synthetic AAV serotypes. Using a p5-7 postnatal AAV injection through the round-window membrane, they tested the efficiency of these overloaded AAVs to induce the expression of functional otoferlin in IHCs of the mouse cochlear explants in cultures as well as in vivo adult mice. They achieved specific expression of otoferlin in ≈30% of all IHCs and partial restoration of hearing in *Otof*^−/−^ mutant mice. These results indicate the feasibility of using the AAV vector to package large genes such as *Otof* to restore hearing function (Table 1, Figure 3).

#### 3.1.2. DFNA25

Mutations in the *SLC17A8* gene coding VGLUT3 cause autosomal dominant deafness linked to auditory synaptopathy. Null mice, with a targeted deletion of exon 2 of the *Slc17a8* gene, displayed an absence of acoustic-stimuli-induced ABRs, while ABRs induced by electrical stimuli were preserved, together with intact OAEs [33,34]. A successful restoration of hearing was demonstrated in this *Slc17a8*-null mouse model by reinstating the expression of Vglut3 via postnatal AAV-mediated delivery, illustrated by the restoration of synaptic transmission and hearing [36]. A recent study showed that AAV8 expressing Vglut3 in the cochleae of 5-, 8-, and 20-week-old Vglut3-null mice resulted in exogenous expression of Vglut3 in all IHCs and successful restoration of hearing for at least 12 weeks via canalostomic injection of AAV-*Vglut3* [106] (Table 1).

DFNA25 patients harboring mutations in the *SLC17A8* gene [33,38,39] exhibited progressive sensorineural hearing loss at high frequencies, and this also was characterized as synaptopathy [33,39]. Thus far, however, AAV-mediated gene transfer to correct mutated sequences and to rescue hearing in DFNA25 rodent models has not been attempted.

#### 3.1.3. DFNB93

Human pathological mutations in the *CABP2* gene have been shown to cause moderate-to-severe, non-syndromic autosomal recessive hearing impairment DFNB93 characterized as auditory synaptopathy [48,49,50,52]. A recent interesting study showed the efficiency of round-window membrane injection of AAV2/1- and AAV-PHP.eB-mediated expression of *CABP2* in IHCs of P5-7 postnatal *Cabp*^−/−^ mice in restoring IHC Cav1.3 function and improved hearing of *Cabp*^−/−^ mice [51] (Table 1, Figure 3).

### 3.2. Hearing Restoration in Syndromic Auditory Neuropathy

#### 3.2.1. Charcot–Marie–Tooth

Charcot-Marie-Tooth (CMT) type 1A (demyelinating neuropathies) is caused by a *PMP22* gene duplication. The 1.4 Mb tandem intra-chromosomal duplication on chromosome 17p11.2-p12 produces three gene copies, each translated into PMP22 protein [61,107,108,109]. Gene-therapeutic approaches to treat CMT1A have been designed to reduce *PMP22* overexpression at the DNA or mRNA level. For this purpose, an *RNA-interference (RNAi)* [110] AAV2/9 vector expressing murine *PMP22*-targeting shRNA [111] and miR-318-downregulated *PMP22* mRNA [112] have been tested in mouse and rat CMT1A models. These therapeutic approaches normalized MPZ and PMP22 protein levels and improved myelination, function, locomotor activity, and electrophysiological parameters [111,112,113,114]. Furthermore, subcutaneous administration of *PMP22-*targeting antisense in a *CMT1A* rat also reduced the mRNA levels of *Pmp22* and improved functional and morphological abnormalities of CMT1A rodent models in a dose-depended manner [115]. However, as for the RNAi technique, antisense therapy requires repeated dosing. CRISPR/Cas9-mediated deletion of the TATA-box promoter of the *PMP22* gene in mice using non-viral intraneural injections also downregulated *Pmp22* mRNA and improved nerve pathology [116]. However, off-target effects of gene editing approaches remain a concern, and mRNA editing techniques such as spliceosome-mediated RNA trans-splicing may be an alternative approach [117].

Finally, supplementation of neurotrophin-3 (NT-3), a neurotrophic factor crucial for Schwann-cell autocrine survival and regeneration, has been proposed to treat CMT1A [118]. Subcutaneous administration of the NT-3 peptide in nude mice harboring CMT1A xenografts, Trembler^J^ mice with a peripheral myelin protein 22-point mutation, and CMT1A patients resulted in improved axonal regeneration in animal models for CMT1A and provided beneficial effects in patients [119]. Subsequently, the same lab showed that injection of AAV1 packaged *NT-3* cDNA into muscle can act as a secretory organ for widespread distribution of *NT-3* in Trembler^J^ mice with demyelinating CMT. This therapeutic approach raised measurable NT-3 secretion levels in blood sufficiently to provide an improvement in motor function, histopathology, and electrophysiology of peripheral nerves of *AAV1-NT-3* cDNA-treated Trembler^J^ mice [120]. These studies of the intramuscular delivery of rAAV1 NT-3 may serve as a template for other nerve diseases involving impaired nerve regeneration. Currently, AAV1 carrying the human NT-3 cDNA scAAV1.tMCK.NTF3 is in a phase I/IIa clinical trial (NCT03520751) using bilateral intramuscular injections in CMT1A patients (Table 1, Figure 3).

Despite the promise of gene therapeutic tools designed to treat CMT1A, their effects on hearing have not yet been assessed.

#### 3.2.2. Autosomal-Dominant Optic Atrophy

It is well known that haploinsufficiency is responsible for isolated DOA, whereas dominant-negative or deleterious gain-of-function types might be responsible for DOA*plus* [121]. Thus, increasing OPA1 expression represents a promising therapeutic approach to treat OPA1-associated diseases. One of these gene therapy approaches is to increase *OPA1 gene* expression at the DNA level. To do so, Sarzi et al. [122] explored the possibility of restoring visual function by intravitreal injections of an AAV2 carrying the human variant #1 OPA1 cDNA, which gives rise to both the long and short OPA1 isoforms. Their results showed that AAV2-mediated WT *OPA1* supplementation therapy might be sufficient to prevent retinal ganglion cell degeneration, although without rescuing visual function [122]. Recently, Jüschke et al. [123] identified a novel OPA1 mutation, c.1065+5G>A, in patients with DOA. This mutation leads to the skipping of OPA1 exon 10 and reducing the OPA1 protein expression by ≈50%. Proper OPA1 function depends, however, on the fine balance of different L- and S-OPA1 isoforms. These authors proposed a promising strategy to convert misspliced OPA1 transcripts into correctly spliced OPA1 transcripts and thus increase the fraction of functional OPA1 transcripts without changing the processing of isoforms. To this end, they engineered U1 splice factors retargeted to different locations in OPA1 exon 10 or intron 10. They showed that application of U1 designed to bind to intron 10 at position +18 led to significant silencing of the effect of the mutation (skipping of exon 10) and increased the expression level of normal transcripts in DOA-patient-derived fibroblasts [123]. This study provides a proof-of-concept for the feasibility of splice-mutation correction as a treatment option for DOA.

Another potential genetic therapeutic option for DOA could be CRISPR–Cas9 gene editing. Using this technique, Sladen et al. [124] successfully achieved correction of an OPA1 c.1334G>A: p.R445H mutant in 57% of isolated DOA-patient-derived pluripotent stem cells (iPSCs). Correction of OPA1 led to restoration of mitochondrial homeostasis, network and basal respiration and ATP production, and reduced susceptibility to apoptotic stimuli in patients’ iPSCs (Table 1, Figure 3).

Altogether, these promising studies pave the way for exploring gene therapy for auditory functional changes in mouse models carrying human *OPA1* mutations.

#### 3.2.3. Leber Hereditary Optic Neuropathy (LHON)

Gene therapies have been designed to treat LHON, consistent with compensation of the mitochondrial complex 1 defect. This approach is based on delivering a functional WT gene ND4 to the nucleus of retinal ganglion cells and then importing it into the mitochondria by adding a mitochondrial targeting sequence to restore respiratory chain activity [125,126]. This strategy has been tested in several rodent LHON models via AAV-mediated intravitreal gene delivery. The different teams showed that intravitreal injection was safe and presented no ocular complications related to the treatment itself. They also showed mitochondrial internalization of AAV, together with the expression of its genetic content and the complementation of the pathogenic phenotype [127,128,129,130,131,132,133] (Table 1, Figure 3).

**Table 1:** Gene therapies for genetic synaptopathies and neuropathies that have been discussed in this review.

Diseases	Defective Genes	Therapeutic Strategies	Benefic Effects	Clinical Trials
DFNB9	*OTOF*	AAV-synaptotagmin 1 [100]	Embryonic inner ear and organotypic culture: Failed to rescue Ca^2+^-influx-triggered exocytosis	DB-OTO phase 1/2 clinical trial in pediatric patients
		Dual AAV-*Otof* [103]	P10-RWM injection Total and sustained rescue ABR threshold shifts Amplitude wave I: 39% of the WT (P10injection), 50% of the WT (P17 injection) Ribbon number twice higher> non treated, but <WT	
		Single overloaded AAV-*Otof* [105]	P5-7 RWM injection: Expression of otoferlin in 30% of IHCs Partial restoration of hearing Poor preservation of wave I	
DFNA25	SLC17A8	AAV1- *Slc17a8* [36]	P1-P2 RW injection: 100% recovery ABR thresholds 40% sustained ABR recovery	
		AAV8- *Slc17a8* [106]	5 w, 8 w, and 20 w canalostomic injection: 5 w injection: restore Vglut3 expression and hearing Partially restore the number of synapses 8 w injection: partial rescue of hearing 20 w injection: rescue less than 50% of ABR threshold	
DFNB93	CABP2	AAV2/1 and PHP.eB-*CABP2* [51]	P5-7 RW injection: Improve at least 20dB in all frequencies in 67% of the injected mice	
CMT	MPZ PMP22	*RNA-interference (*RNAi)** [110] AAV2/9 -*Pmp22* shRNA [111] miR-318 [112] CRISPR/Cas9 [116] *PMP22* *antisense* [115]	Intraneural injections: Normalize MPZ and PMP22 protein levels Improve myelination, function, locomotor activity, and electrophysiological parameters Subcutaneous injection: Reduce the mRNA levels of *Pmp22*, improve functional and morphological abnormalities of CMT1A	
		NT-3 supplementation [119]	Subcutaneous injection: Improve axonal regeneration	
		AAV1-*NT*-*3 cDNA* [120]	Intramuscular injection: Improve motor function, histopathology, and electrophysiology of peripheral nerves	phase I/IIa clinical trial (NCT03520751)
DAO	OPA	AAV2-*OPA1* [122].	Intravitreal gene delivery Reduce retinal ganglion cell degeneration without rescuing an efficient visual acuity	
		U1 splice factors [123] (bind to intron 10 at position +18 of OPA1)	In vitro: patient-derived and control fibroblasts Silence the effect of the mutation, increase the expression level of normal transcripts	
		CRISPR/Cas9–iPSCs (c.1334G>A: p.R445H) [124]	In vitro: Restore mitochondrial homeostasis, re-establish the mitochondrial network, basal respiration, and ATP production levels	
LHON	Mt DNA TMEM126A	rAAV5-NDI1 [128]	Stereotaxic injections: infusion into the optical layer of the SC Rescue vision loss induced by complex I deficiency	
		AAV2-NDI1 [131]	Intravitreal gene delivery: Mitochondrial internalization of AAVV Reduce RGC death and optic nerve atrophy Preserve retinal function (manganese, Mn2 þ)-enhanced magnetic resonance imaging (MEMRI) and optokinetic responses	Phase 1 clinical trial of scAAV2-P1ND4v2 of ND4-LHON (NCT02161380)
		AAV2-ND4 [125,127,129,133]	Restore the activity of the respiratory chain and rescuing retinal ganglion cell degeneration	Phase 3 pivotal clinical study of rAAV2/2-ND4: REFLECT (NCT03293524)

Abbreviation used in the table: RWM: round window membrane. w: weeks.

## 4. Conclusions

This review of the literature described pathogenic mechanisms mediating genetic ANSD, as well as genetic therapies that are currently in development. The discovery of the gene responsible for ANSD ushered in a new and exciting time for drug discovery and therapeutic genetic modulation. New discoveries are continuing to drive innovation in the development of innovative treatments for both non-syndromic and syndromic ANSD. In recent years, substantial progress has been made in developing gene therapeutic tools to regenerate auditory synapses and neurons, or to replace defective genes through gene therapies. Nevertheless, regardless of advances in capabilities for gene delivery, the complex nature of regeneration and repair processes and the wide range of molecular and cellular targets underscore the need for precisely controlled systems capable of delivering a wide range of biomaterials such as genes, siRNAs, RNAs, and DNAs.

One exciting approach relies on the use of genome-editing technologies based on programmable nucleases, including CRISPR–Cas9 [134]. These new technologies allow us to remove or correct deleterious mutations or insert protective mutations in diseased cells and tissues. The injection of CRISPR–Cas9 complexes into the ears of neonatal Beethoven mutant mice improved auditory function [135], thus providing a potential therapeutic option for deaf patients carrying monogenic mutations. CRISPR/Cas9-mediated deletion of the TATA-box promoter of the *PMP22* gene in a Charcot–Marie–Tooth mouse model using non-viral intraneural injections also downregulated *PMP22*mRNA and improved nerve pathology [116]. However, off-target effects of gene editing approaches remain a concern.

An alternative approach to correcting gene mutations is using spliceosome-mediated RNA *trans*-splicing, or SMaRT. This technique targets the mRNA sequence to correct the mutations. The proof-of-concept of SMaRT has already been established in several models of genetic diseases caused by recessive mutations [117]. This innovative technology has not yet been investigated in the inner ear but offers hope of a single treatment for restoring hearing in patients carrying recessive gene mutations.

The bench-to-bedside transition for AAV-mediated gene therapy took its first steps in 2008, when the efficacy of gene therapy was demonstrated to treat Leber congenital amaurosis. Three successful clinical trials were completed regarding the safety of subretinal injection of 65 kDa retinal pigment epithelium-specific protein (RPE65) expressed by an AAV vector for Leber congenital amaurosis [136,137,138,139]. Published studies of clinical trials of genes designed to compensate for the mitochondrial complex 1 defect with the functional wild-type gene with intravitreal injection of AAV2-ND4 in ND4-LHON patients reported clinically meaningful beneficial effects beyond the expected natural history of the disease [125]. AAV2-ND4 successfully restored the activity of the respiratory chain and rescued retinal ganglion cell degeneration [125].

These trials have paved the way for the first FDA-approved gene therapy products to treat ANSD. DB-OTO, a lead gene therapy product candidate directed by Decibel Therapeutics company to treat otoferlin mutation-induced DFNB9, has received clearance from the U.S. FDA to initiate a phase 1/2 clinical trial in pediatric patients [140]. OTOF-GT, a lead gene therapy candidate developed by Sensorion biotech, has also been granted rare pediatric disease designation from the U.S. FDA [141] for treating pediatric DFNB9 patients. To date, there have been multiple clinical trials studying AAV-mediated gene therapy in optic neuropathies. However, there have been no trials involving auditory neuropathies, although the gene therapy of monogenic disease using AAV has become feasible. The discrepancy between the progress of optic and auditory neuropathy gene therapies has mostly been attributed to the earlier preclinical success and the increased accessibility for treatments of the eye relative to the cochlea. In addition, heterogeneity is the major challenge in the treatment of genetic ANSD, as several factors affect treatment efficacy, such as therapeutic window, targets, targeting molecules, and protein function.

Future therapies to restore synaptic transmission or to regenerate auditory nerve fibers must consider multiple targets that account for the complexity of disease-causing factors and pathogenetic mechanisms. Looking back on the historical, functional, and molecular achievements made in this field, each were made possible by technological developments defining a new epoch. In order to overcome the somewhat static current status in terms of clinical trials, we now need to refine the protocol of these trials and search for more predictive animal models of deafness on which they are based. Additionally, there is also a need to develop more reliable clinical diagnostic tools for early identification of ANSD, as well as to carefully evaluate the degree of degeneration of auditory synapses and nerve fibers. Clinical trials of biologic agents to treat ANSD need valid clinical outcome measures and biomarkers.

## Data Availability

All data analysed during this study are included in this published article.
